# Clinical features of pituitary carcinoma: analysis based on a case report and literature review

**DOI:** 10.3389/fendo.2024.1440247

**Published:** 2024-10-31

**Authors:** Yongxiang Yang, Wanlin Liang, Kexia Fan, Tao Yang, Jingmin Cheng

**Affiliations:** ^1^ Department of Neurosurgery, The General Hospital of Western Theater Command, Chengdu, China; ^2^ School of Clinical Medicine &The First Affiliated Hospital of Chengdu Medical College, Chengdu, China

**Keywords:** pituitary carcinoma, metastasis, temozolomide, radiotherapy, Ki-67, p53

## Abstract

**Introduction:**

Pituitary carcinoma (PC) is an extremely rare tumor of the adenohypophysis, which manifests as craniospinal dissemination and/or systemic metastasis. The diagnosis of PC is particularly difficult, as the clinical diagnosis only can be made after the metastasis is found. Owing to the complex diagnostic process and less effective treatments, the clinical prognosis of PC is usually very poor. Hence, it is of great significance to illustrate the diagnosis and treatment course of PC.

**Methods:**

In this case report, we described a 48-year-old male patient who was diagnosed with pituitary adenoma (PA) initially and then was diagnosed with PC eventually after spinal cord metastasis was found, and we illustrated the treatment course as well. Furthermore, we summarized all the published case reports until now and provided a comprehensive review of the diagnosis, treatment, prediction, and clinical outcome of PC.

**Results and Conclusions:**

We found that most PC patients had adrenocorticotropic hormone/prolactin (ACTH/PRL)-secreting tumors, Ki-67 ≥ 10%, and P53 positivity, which may have the potential to predict the transformation from PA to PC; surgery excision combined with temozolomide (TMZ) and radiotherapy is helpful to prolong the survival of PC patients.

## Introduction

Pituitary adenomas (PAs) originate from the endocrine cells of the anterior pituitary, which are the second most common intracranial tumors and account for approximately 15% of intracranial neoplasms (1, 2). Most PAs are regarded as benign, growing slowly and rarely invading into the surrounding tissues. Nevertheless, 20% to 25% of PAs grow invasively and even aggressively infiltrate the dura mater basilar bone, cavernous sinuses, or sphenoid sinus (3, 4). In extremely rare cases, some PAs show malignant biological behaviors such as craniospinal dissemination and/or systemic metastasis, which can be called pituitary carcinomas (PCs) (5). PCs had been defined by the 2017 World Health Organization (WHO) classification of PAs and the 2017 European Society of Endocrinology (ESE) guidelines on aggressive PAs and PCs based on the presence of metastasis (5–7).

PCs account for 0.1% to 0.2% of PAs more or less; the clinical diagnosis of this rare disease is particularly difficult because it only can be made when primary PAs present and delayed metastasis in the brain, spinal cord, or other distant organs is found (8, 9). Moreover, the clinical manifestations of PCs are related to the functional state of the primary lesion and the location and size of metastatic lesions, which are highly variable and difficult to identify from other non-specific presentations (6, 10). Herein, the early diagnosis of PCs is extremely challenging. Currently, it is almost impossible to predict the clinical features and outcomes according to existing histological results including invasiveness, cellular pleomorphism, nuclear type, mitosis, and necrosis (11). Owing to the difficulty of early diagnosis of PCs and the absence of reliable prognostic criteria or pathological markers of PCs, the treatment and management of PCs are facing many challenges. At present, most PC patients survive less than 1 year after diagnosis, as the existing treatments including surgery, chemotherapy, radiotherapy, and immunotherapy are usually partially effective or not effective (12, 13). Hence, the clinical prognosis of PCs is very poor, and it is of great significance to illustrate the diagnosis and treatment course of PCs.

Up to now, the knowledge about PCs almost entirely comes from case reports or case series, which is far from enough. Although the ESE published clinical practice guidelines for the management of aggressive PAs and PCs in 2017 (5), its guiding value for clinical practice is limited. Accordingly, it is very meaningful to illustrate the diagnosis and treatment process of PCs and review the literature. This study described a 48-year-old male patient who was diagnosed with an atypical pituitary tumor initially and then was diagnosed with PCs eventually after spinal cord metastasis was found. Furthermore, all the published works of literature about PCs including case reports and reviews until now were summarized to review the epidemiology and diagnosis, clinical characteristics, available predictive markers and potential factors implicated in their aggressiveness, current and emerging therapeutic approaches, and outcomes of PCs. The aims of this study were to explore the key clinical aspects of PCs, further enrich the knowledge about PCs, and improve the clinical outcomes of this rare disease.

## Methods

First, we retrospectively described the diagnosis, treatment, and outcome of a 48-year-old male patient with PC with spinal cord metastasis. Then, we comprehensively searched all the published English literature with the keyword “pituitary carcinomas” through PubMed and summarized the clinical data of the patients in the literature, including gender, age of onset, pathological results, site of metastasis, interval time of metastasis, treatment method, and final clinical outcome. In the clinical data of patients reported in the literature, we focused on analyzing the correlation between the index of P53 and Ki-67, the interval time of pituitary metastasis, and the clinical outcome of PC patients. In addition, we analyzed the correlation between the treatment methods including surgery, radiotherapy, chemotherapy, and the clinical outcome of PC patients. Finally, we comprehensively summarized the diagnosis, prognosis, treatment, and final outcome of PC by reviewing the literature. In the analysis process, we analyzed the enumeration data (P53, Ki-67, and 2-year survival state) by chi-squared test using SPSS version 18.0 software (SPSS Inc., USA), and two-tailed p < 0.05 was considered statistically significant.

## Case description

**Figure 1:**
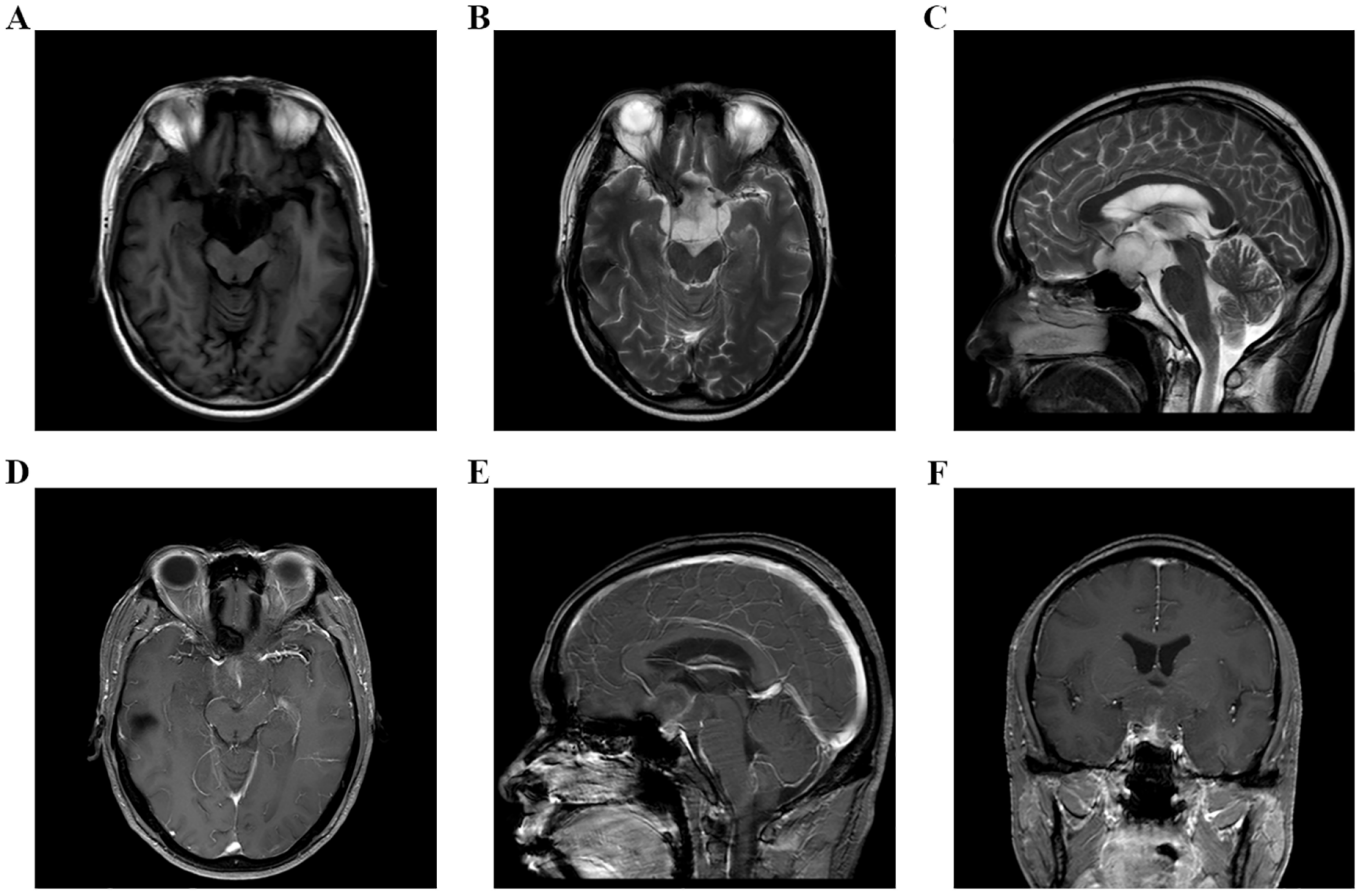
**(A–F)** MRI examination of head on September 8, 2023, indicated that there was an occupying lesion in the sellar area and suprasellar cisterna, the size was 4.4 × 3.9 × 2.6 cm, and the grade was Knosp3.

**Figure 2:**
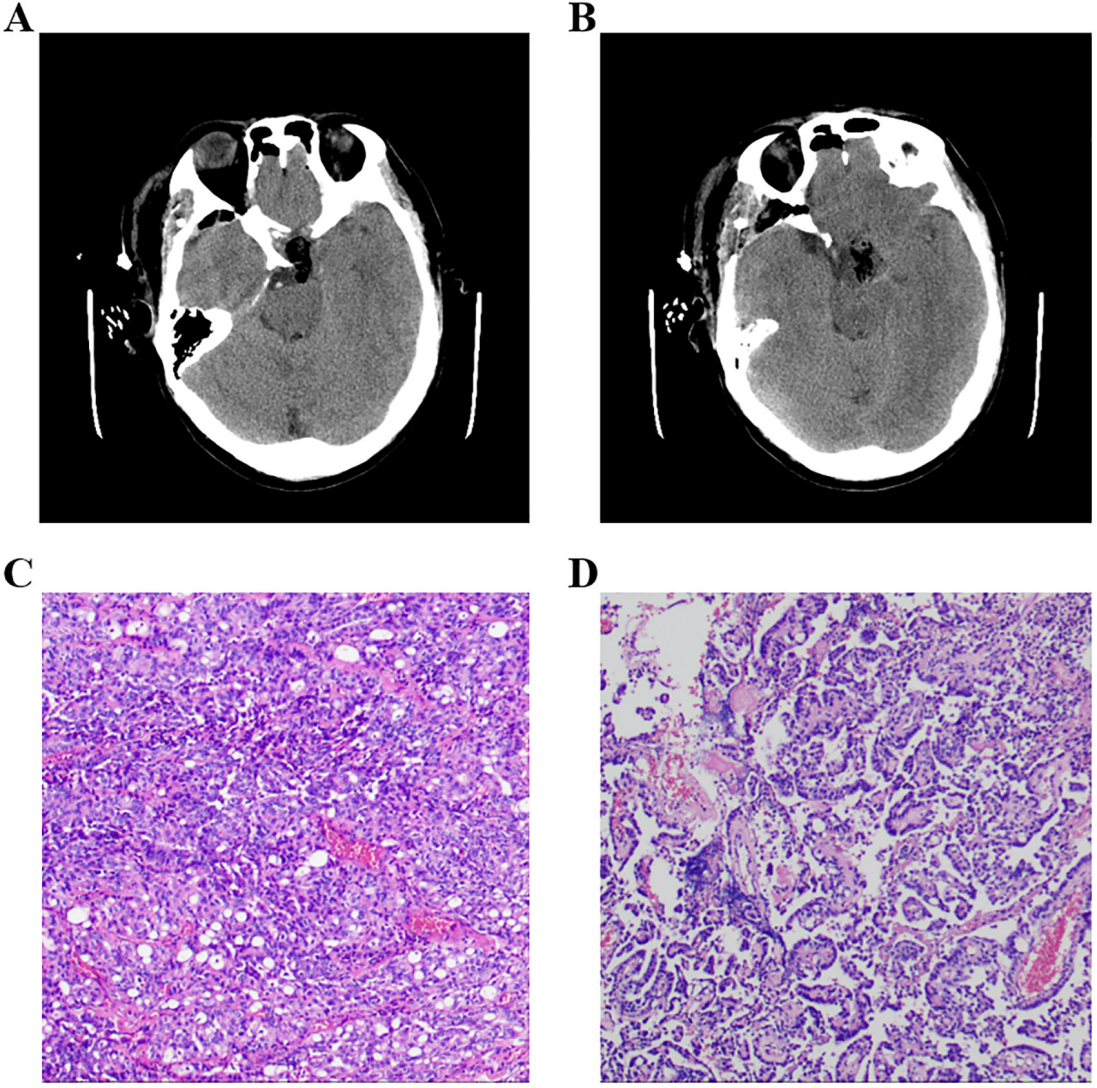
**(A, B)** Postoperational CT examination of the head. **(C, D)** Pathology results of the tumor, and the immune markers were as follows: ACTH(−), CD56(+), CK(+), CK20(−), CK8/18(+), CgA(−), FSH(−), GFAP(−), GH(−), Ki-67(+,3-5%), LH(−), NSE(+), P53(+), P63(−), PIT-1(−), PR(−), PRL(−), S-100(+), SF-1(−), Syn(−), TSH(−), TTF-1(−), Tg(−), Villin(−), Vim(+), and WT-1(+). ACTH, adrenocorticotropic hormone; PRL, prolactin; GH, growth hormone; TSH, thyroid-stimulating hormone; FSH, follicle-stimulating hormone; LH, luteinizing hormone.

**Figure 3:**
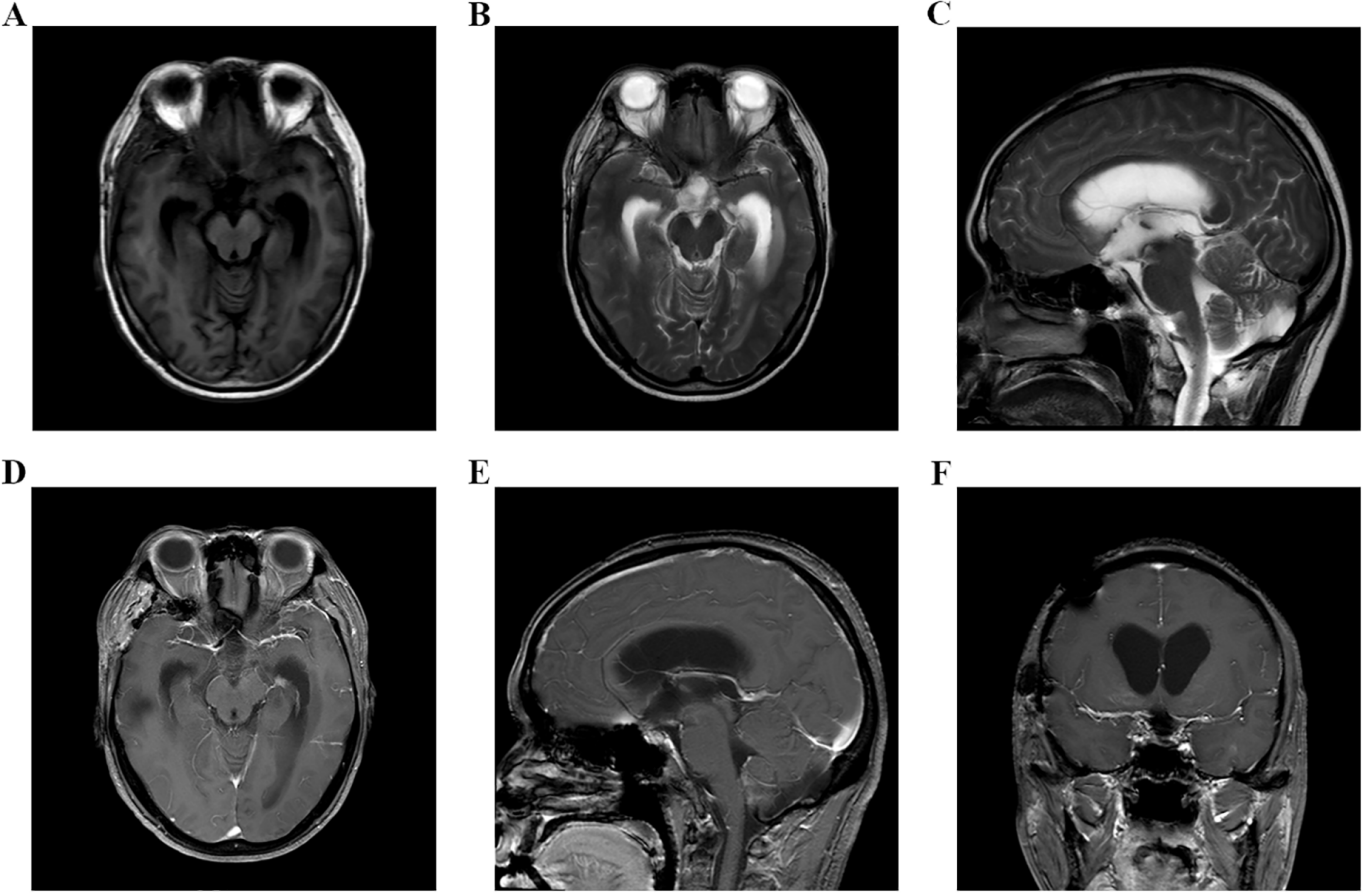
**(A–F)** Head MRI examination on November 21, 2023, indicated that there was no tumor recurrence in the sellar area, but the third ventricle and bilateral lateral ventricles were dilated.

**Figure 4:**
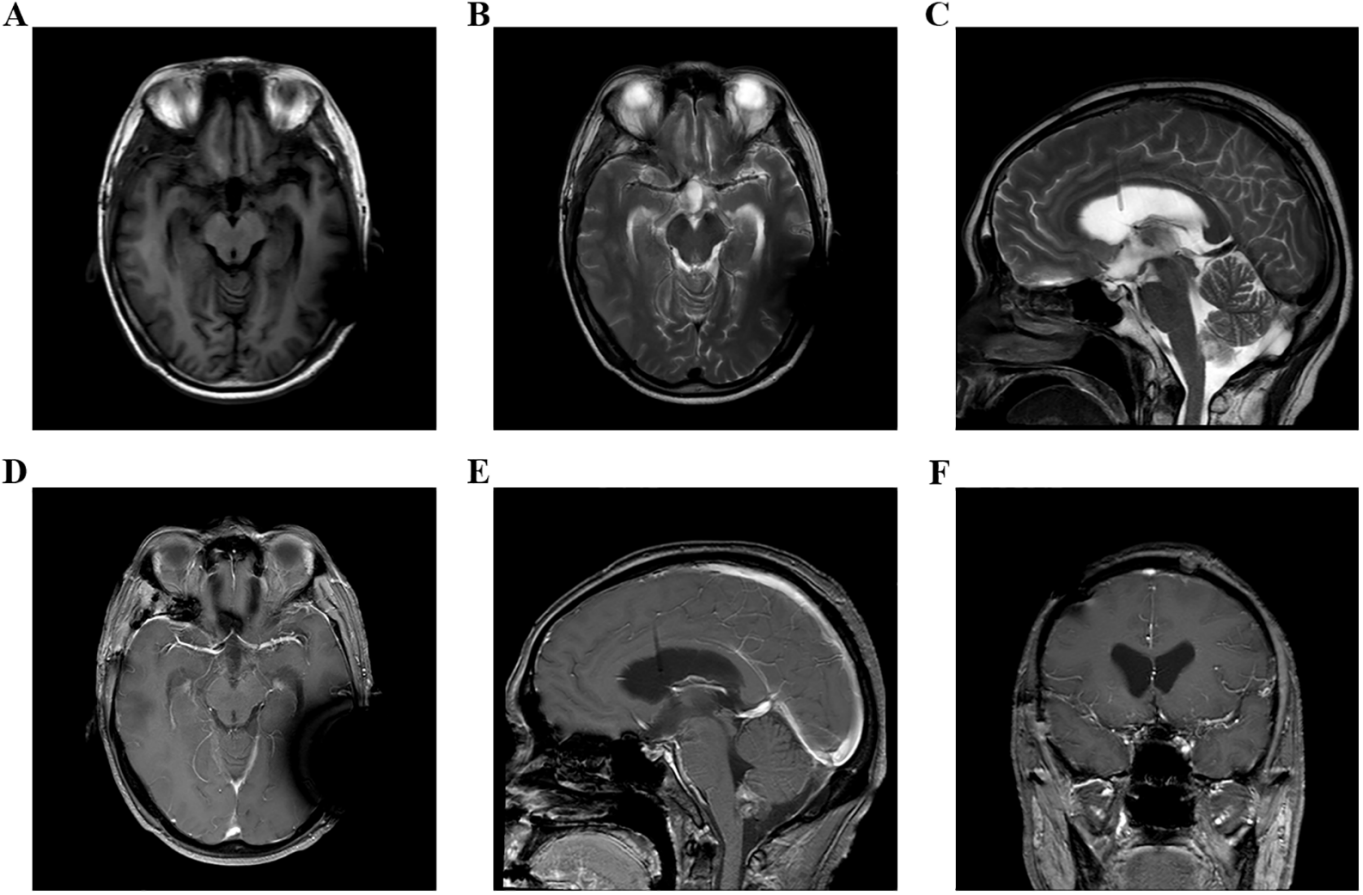
**(A–F)** Head MRI examination on December 9, 2023, indicated that the volume of third ventricle and bilateral lateral ventricles were smaller than before.

**Figure 5:**
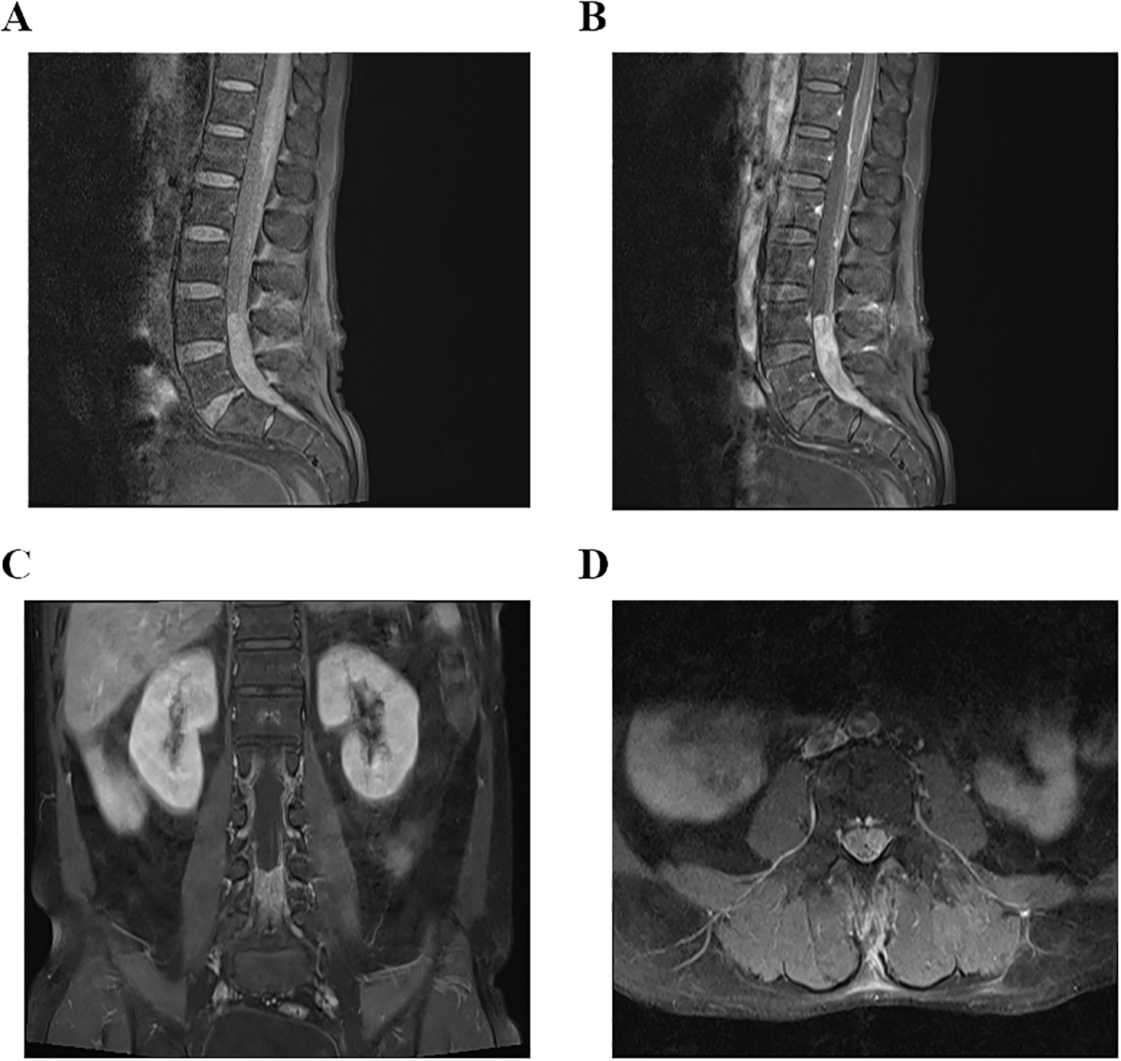
**(A–D)** MRI examination of lumbar spine on December 15, 2023, indicated that there was an occupying lesion in the L4–S1 intraspinal area, and the size was 1.5 × 1.4 × 9.2 cm.

A 48-year-old male patient came to our hospital and sought diagnosis and treatment on September 7, 2023, mainly due to “headache, bilateral blurry vision and narrowed visual field for more than 6 months”. The patient began to have headaches and blurry vision in both eyes in March 2023 without obvious cause, and his situation did not improve after treatment in the ophthalmology department of other hospitals. Later on, the patient manifested a gradual aggravation of bilateral blurry vision and began to have narrowed visual field little by little, and his condition did not improve after searching for a cure in the ophthalmology departments of other hospitals many times. Then, the patient received a head MRI examination at another hospital on September 1, 2023; an occupying lesion was found in the sellar area and suprasellar cisterna, and the diagnosis was considered to be a pituitary tumor, craniopharyngioma, epidermoid cyst, or other neoplastic lesions. In order to seek further diagnosis and treatment, the patient came to our hospital and was admitted to our neurosurgery department on September 7, 2023. The patient was in good health previously and had no family history of tumors or other hereditary diseases. Physical examination revealed that the patient had binocular vision and temporal hemianopsia. The MRI examination of the head on September 8, 2023, indicated there was an occupying lesion in the sellar area and suprasellar cisterna, the size was 4.4 × 3.9 × 2.6 cm, and the diagnosis was considered to be a pituitary tumor, craniopharyngioma, epidermoid cyst, or other neoplastic lesions (**Figure 1**). As the lesion invaded bilateral cavernous sinuses and crossed the internal carotid artery, the grade of this PA was Knosp3. Laboratory test results showed that the serum levels of prolactin (PRL), adrenocorticotropic hormone (ACTH), growth hormone (GH), follicle-stimulating hormone (FSH), luteinizing hormone (LH), IGF-1, and thyroid-stimulating hormone (TSH) were all normal. Considering the medical history, clinical symptoms, physical signs, and examination results of the patient, we made a possible diagnosis of a pituitary tumor, craniopharyngioma, or other tumor lesions. After communicating with the patient and his family members about the condition and treatment plan, they asked for a “craniotomy to remove intracranial tumor” and signed the informed consent. Then, a craniotomy sellar tumor resection was successfully performed on September 12. 2023. The postoperative pathological diagnosis was a non-functional PA; the immune markers were as follows: ACTH(−), CD56(+), CK(+), CK20(−), CK8/18(+), CgA(−), FSH(−), glial fibrillary acidic protein (GFAP) (−), GH(−), Ki-67(+,3-5%), LH(−), neuron specific endase (NSE) (+), P53(+), P63(−), PIT-1(−), progesterone receptor (PR) (−), PRL(−), S-100(+), SF-1(−), Syn(−), TSH(−), TTF-1(−), Tg(−), Villin(−), Vim(+), and WT-1(+) (**Figure 2**). The patient’s headache symptoms were relieved, bilateral vision and visual field improved gradually after the operation, and he was discharged on September 23, 2023. On November 18, 2023, the patient was readmitted to our hospital due to “headache combined with blurred vision and vomiting for 2 weeks”. Physical examination revealed that the patient had blurred vision. A head MRI examination on November 21, 2023, indicated there was no tumor recurrence in the sellar area, but the third ventricle and bilateral lateral ventricles were dilated (**Figure 3**). Cerebrospinal fluid examination showed that the pressure was 350 mmH_2_O, and the biochemical/routine index was normal. The diagnosis was considered to be hydrocephalus, and a ventriculoperitoneal shunt surgery was performed on December 7, 2023. The patient’s headache symptoms were relieved, and bilateral vision improved gradually after the operation. A head MRI examination on December 9, 2023, indicated that the volume of the third ventricle and bilateral lateral ventricles was smaller than before (**Figure 4**). On December 12, 2023, the patient began to manifest severe pain in the left hip and left lower limb. An MRI examination of the lumbar spine on December 15, 2023, indicated that there was an occupying lesion in the L4–S1 intraspinal area, the size was 1.5 × 1.4 × 9.2 cm, and the diagnosis was considered to be a metastatic tumor (**Figure 5**). Considering the medical history, clinical symptoms, physical signs, and examination results of the patient, we made a diagnosis of PC with spinal cord metastasis. After communicating with the patient and his family members about the therapeutic strategy for PC with spinal cord metastasis, they refused to accept further treatments including surgery, radiotherapy, and chemotherapy, and he was discharged on January 11, 2024. In the follow-up survey, we learned that the patient underwent intraspinal metastatic resection at another hospital on January 29, 2024, and the postoperative pathological diagnosis was considered to be a tumor metastasized from the PA; his immune markers were as follows: GFAP(partial+), Oligo-(2−), EMA(dot +), P53(in +), Syn(+), CgA(−), SOX-2(+) and SOX-2(−), ATRX IDH-1(+), H3 K27M(−), INI1 (without missing), and Ki-67 (MIB) (+5% to 10%). Then, the patient received temozolomide (TMZ) therapy for 3 months, but his result was not satisfactory; his eyesight was becoming worse, and the muscle strength of both lower limbs gradually decreased.

## Literature summary and analysis results

**Figure 6:**
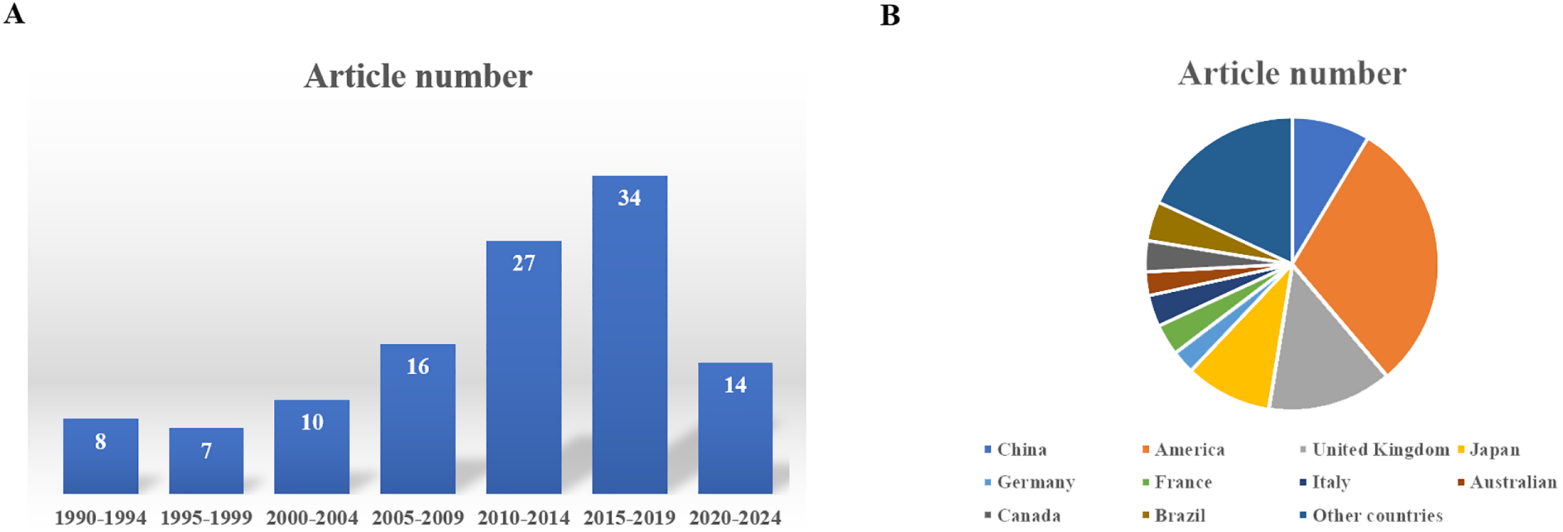
**(A)** The distribution of the publishing time of these articles. **(B)** The distribution of the country of authors of these articles.

“PubMed” was searched using the keyword “pituitary carcinoma” until the time of May 25, 2024. A total of 116 articles were reviewed, and 128 PC patients were included in the literature eventually (8, 13–124). An additional 41 articles were excluded from this summary due to the inaccessibility of the full article. Most of the articles were published at the time interval of 2010–2019 and in the USA, China, and the United Kingdom (**Figure 6**). The clinical data of the included patients are illustrated in **Table 1**. Among the 128 PC patients, 64 were men and 64 were women, and their average age at diagnosis was 48.2 years and ranged from 9 to 75 years. The average time interval from the diagnosis of PAs to PCs was 9.7 years within 0–31 years. The most common pathological type of PC was ACTH with 50 cases (39.1%), followed by PRL with 24 cases (18.8%) and non-functional PC with 25 cases (19.5%). Moreover, there were seven patients who had tumors that secreted two hormones. The most common metastasis site was intracranial with 48 patients (37.5%), followed by spinal metastasis with 29 patients (22.7%), liver metastasis with 18 patients (14.1%), cervical lymph node with 10 patients, and bone metastasis (7.8%) with nine patients (7%). In addition, there were 38 PC patients who had multiple metastatic sites. The treatment of PCs is usually multimodal including surgery, radiotherapy, and chemotherapy. The average surgery time of reviewed patients was 2.7, and 17 patients received five to eight surgical procedures throughout the course of treatment. The transsphenoidal surgical approach was applied in 50 patients (41.3%), of which the endoscopic approach was used in 40 patients (80%). The transcranial approach was applied in 27 patients (22.3%), and the transcranial combined with transsphenoidal approach was applied in 44 patients (36.4%). Radiation therapy was employed in the treatment of 112 patients (87.5%), and chemotherapy was given to 61 patients (47.7%). Among the patients who received chemotherapy, 37 patients (60.7%) were treated with TMZ. Of the 128 patients, 63 patients (49.2%) died as reported, 42 patients (32.8%) were alive at the time of publication, and 23 patients (18.0%) were ambiguous in their survival status. As for the deaths reported, 37 patients died within 1 year of diagnosis, and 18 patients died within 4 years of diagnosis. The average survival time since the diagnosis of PC was 10.5 months, ranging from 6 months to 18 years. The 2-year survival rate of PC patients who received TMZ treatment was increased by a minor degree compared to that of patients who did not receive TMZ therapy, but without statistical significance (**Table 2**). The Ki-67 and P53 were detected in 43 and 26 patients, respectively; the rate of Ki-67 ≥ 10% and P53 positive both increased significantly “after metastasis” than “before metastasis” (**Table 3**).

**Table 1:** Clinical characteristics of pituitary carcinoma patient cases.

Variable	Value
Gender (n, %)	
Male	64 (50%)
Female	64 (50%)
Average age at PC diagnosis	48.2 years
Pathological type of PC (n, %)	
ACTH	50 (39.1%)
PRL	24 (18.8%)
GH	13 (10.2%)
TSH	3 (2.3%)
FSH	3 (2.3%)
LH	2 (1.6%)
Non-functional	25 (19.5%)
The site of metastasis (n, %)	
Intracranial	48 (37.5%)
Spinal	29 (22.7%)
Liver	18 (14.1%)
Cervical lymph nodes	10 (7.8%)
Bone	9 (7.0%)
Lung	6 (4.7%)
Endolymphatic sac	5 (3.9%)
Orbit	3 (2.3%)
Average interval time from PT to PC diagnosis	9.7 years
Average number of surgery	2.7
Number of surgical patients (n, %)	121 (94.5%)
Transsphenoidal approach	50 (41.3%)
Transcranial approach	27 (22.3%)
Transcranial and transsphenoidal approach	44 (36.4%)
Radiation therapy (n, %)	
Yes	112 (87.5%)
No	16 (12.5%)
Chemotherapy (n, %)	
Yes	61 (47.7%)
Temozolomide treated	37 (60.7%)
No	49 (38.3%)
Mortality (n, %)	
Alive at the time of publication	42 (32.8%)
Death	63 (49.2%)
Average time from PC diagnosis to death	10.5 months
Not reported	23 (18.0%)

ACTH, adrenocorticotropic hormone; PRL, prolactin; GH, growth hormone; TSH, thyroid-stimulating hormone; FSH, follicle-stimulating hormone; LH, luteinizing hormone; PCs, pituitary carcinomas; PAs, pituitary adenomas.

**Table 2:** Comparison of survival state between those treated with TMZ and those without TMZ.

Variable	With TMZ (n = 30)	Without TMZ (n = 52)	χ^2^ value	p-Value
**Survival state**			0.928	0.335
Alive at 2 years (n, %)	13	17		
Died at 2 years (n, %)	17	35		

Patients without reported survival status or adequate follow-up were excluded from analysis.

TMZ, temozolomide.

**Table 3:** The Ki-67 and P53 index before and after the metastasis.

Variable	Before metastasis	After metastasis	χ^2^ value	p-Value
**Ki-67 (n, %)**			16.125	<0.001^*^
<10%	25	7		
≥10%	18	36		
**P53 (n, %)**			5.65	0.017^*^
Positive	17	24		
Negative	9	2		

p < 0.05 represents statistically significant (marked with * and bold).

## Literature review and discussions

PAs are relatively common sellar tumors accounting for approximately 15% of intracranial tumors, whereas PCs are exceedingly rare diseases with an incidence of approximately 0.1% to 0.2% of PAs (125, 126). In this case report, we described the diagnosis and treatment course of a PC patient with spinal cord metastasis. We also summarized the case reports/series in the literature and reviewed the clinical features, epidemiology and diagnosis, available predictive markers and potential factors implicated in their aggressiveness, current and emerging therapeutic approaches, and outcomes of PCs.

PCs usually progress from PAs after a very long or short latency period following the diagnosis of original PAs (57, 127). Most PC patients manifest a similar clinical course as PA but with repeated recurrence and delayed metastasis; a few PC patients present rapid malignant behavior, multiple recurrences, and early metastasis (128). The clinical course of our reported patient falls in the latter group, with a manifestation of PC within 6 months after the diagnosis of PA. According to the reviewed literature, the mean latency period between the diagnosis of PA and the identification of PC in the 128 cases is 9.7 years, which is longer than the reported “6 years” in a case series by the University of Texas, MD Anderson Cancer Center. Only 28 patients were diagnosed with PC within 1–3 years of the diagnosis of PA. Moreover, the time of latency period is likely to be related to the hormone-secreting type of PC. Most PCs have endocrine activity and are transformed from ACTH/PRL-secreting PAs, whose average latency period is 9.5 and 4.7 years, respectively (129). The fifth edition of the WHO classifications (2021 World Health Organization Classification of Central Nervous System Tumors and 2022 World Health Organization Classification of Endocrine and Neuroendocrine Tumors) has introduced significant changes to the classification of PAs (130). It is worth noting that the anterior pituitary hormone-secreting cells are categorized into three major groups based on their corresponding transcription factors: PIT1, TPIT, and SF1. In the new WHO classification, the PIT1 group comprises somatotroph tumors, lactotroph tumors, mammosomatotroph tumors, thyrotroph tumors, other mature plurihormonal PIT1-lineage tumors, immature PIT1-lineage tumors, acidophil stem cell tumors, mixed somatotroph, and lactotroph tumors. Corticotroph tumors are categorized in the TPIT group, while somatotroph tumors belong to the SF1 group. Moreover, mammosomatotroph tumors and acidophil stem cell tumors are classified into the PIT1 group, and plurihormonal PIT-1-positive PA was divided into immature PIT1-lineage and mature plurihormonal PIT1-lineage tumors.

The diagnosis of PCs is exceedingly difficult because it only can be made when original malignant PAs are present and there is combined metastasis in the brain, spinal cord, or other distant organs. The absence of histological characteristics of malignancy and the delayed presentation of metastasis make the early diagnosis of PC problematic (131). The majority of PCs originate from functional PAs and are mostly represented by ACTH/PRL-secreting tumors, and a small part of PCs evolve from functional PAs switched from non­functional tumors (8, 57, 132, 133). The transformation from PAs to PCs is usually combined with some clinical features such as abnormal hormone secretion, cranial nerve palsy, neck and back pain, obstructive hydrocephalus, and discordance between biochemical and radiological findings (133). This phenomenon was also observed in our report, as the patient first showed the symptom of hydrocephalus and then manifested severe pain in the left hip and left lower limb. The most common metastasis localizations are intracranial (43.1%) and spinal (37.5%), followed by the liver (13.9%), cervical lymph node (11.1%), and bone (9.1%), and rarely in the lung (4.2%), endolymphatic sac (2.8%), or orbit (1.4%) (8), which is similar with our research results. To search for the metastasis localizations, ESE guidelines recommend performing the examination of CT/MRI and/or FDG/SST­PET/CT when PC patients present with some specific symptoms such as neurological complaints or neck and/or back pain or discordances between biochemical and radiological findings (5). The patient in our case report first presented with hydrocephalus and severe pain in the left hip and left lower limb; he then received a lumbar spine MRI examination and was found to have an occupying lesion in the L4–S1 intraspinal area, and the diagnosis was considered to be PC with spinal cord metastasis ultimately.

Owing to the majority of PCs being transformed from aggressive PAs, it is significant to distinguish aggressive PAs and identify PAs with potential for metastasis using some specific molecular markers. Now, the most commonly reported and intensively studied molecular markers are Ki-67 and P53. Ki-67 labeling index uses the MIB-1 antibody, and its mean level was reported to be 11.9% ± 3.4% in PCs compared to 1.4% ± 0.15% in non-invasive PAs (12, 134, 135). As 35%–61% of PAs and PCs had a Ki-67 index of ≥10% (6, 136–140), this criterion was considered to be indicative of PCs. PC patients with a Ki-67 index of ≥10% were proposed to be malignant potential including clinically aggressive, invasive, and highly proliferative (6). Though many authors considered that PAs with a Ki-67 index of more than 10% should be diagnosed as primary PCs (96, 128), the Ki-67 still could not be established as a prognostic marker due to the lack validation of a large number of clinical data (141). Moreover, Ki-67 is an imperfect marker of malignant potential, as the Ki-67 level variably ranged from 0% to 21.9%, and not all studies showed an association between Ki-67 and invasiveness (142, 143). P53 is encoded by the tumor suppressor TP53 gene, which is another protein implicated in PCs and has the prognostic value for the malignant potential (3, 144) but almost never mutates in PAs. Nevertheless, a part of reported PC cases were without P53 immunopositivity (12, 135, 142), and some authors did not consider P53 to be a regular basis for the prediction of the malignant potential (145). Moreover, some case series reports demonstrate that the use of P53 immunodetection as a prognostic tool is controversial (136, 145–147). A recent ESE survey showed that Ki-67 ≥ 10% was frequently seen in 85% and P53 was positive in 78% of 34 PC patients, indicating these two markers had a strong prognostic value for PCs (5). The 2017 WHO classification of PAs does not recommend using the Ki-67 index and P53 to predict tumor invasion, but these two markers are recommended in the ESE clinical practice guidelines to predict the tumor behavior of PAs (5, 145). In addition, the 2017 and 2022 WHO classifications of PAs both encourage the use of transcription factors to predict the tumor behavior of PAs (129, 145, 148). Herein, it is not advisable to predict the malignant potential of PAs solely based on existing pathological markers Ki-67 and P53, and the combination of clinical investigations, radiological manifestations, and further pathological indexes is very essential. Our research based on the reviewed literature showed that the rate of Ki-67 ≥ 10% and P53 positivity after metastasis were significantly higher than those before metastasis. These results indicated the potential value of Ki-67 and P53 in the prediction of PAs transforming into PCs.

The treatment of PCs is usually multimodal including surgery, radiotherapy, and chemotherapy. Surgical therapy is the mainstay treatment of PCs, which can alleviate acute mass effects with debulking through gross total or subtotal resecting of the sellar tumor (134, 135). The transsphenoidal surgical approach, especially the endoscopic approach, is considered to be the first-line treatment of most PC patients, which allows more extensive resections of tumors invading the cavernous sinus and parasellar structures (149). The transcranial approach may exhibit an advantage of obtaining a near-total tumor resection when the tumor presents with intracranial extension. In some cases, multiple repeated surgeries may be performed to resect recurrence and extension lesions, and locoregional therapeutic surgery should be considered to address metastatic sites if amenable to resection (5, 150). Our reported patient received transcranial surgery, and his bilateral vision and visual field improved gradually after the operation, indicating that the decompression effect on the optic nerve is remarkable. According to the reviewed literature, PC patients underwent 2.7 surgical interventions on average, and the transsphenoidal surgical approach accounted for approximately 41.3% of all initial sellar operations. Radiation therapy is recommended by the ESE guidelines and commonly employed for the primary treatment of PCs, which can be delivered as stereotactic radiosurgery, adjuvant radiation therapy, or fractionated radiation therapy over a 5–6-week course (133). In clinical practice, both stereotactic radiosurgery (SRS) and fractionated stereotactic radiotherapy (FSRT) are being used to obtain good disease control, while the success rate is varied and difficult to assess due to the variation in technique and doses administered in reported cases (151). The rate of long-term tumor control and hormone level normalization of the radiation therapy was reported to be 80%–97% and 40%–70%, respectively (152). The most frequent complication of radiation therapy is hypopituitarism, whose incidence is approximately 30%–60% 5–10 years after the treatment (153, 154). The incidence of other rare complications such as radiation-induced optic neuropathy, cerebrovascular accidents, and secondary tumors is approximately 0%–3% (152, 155, 156). According to the reviewed literature, 87.5% of PC patients had undergone SRS/FSRT. Chemotherapies are frequently used in the treatment of PC, of which the most common one is TMZ. TMZ is an oral alkylating agent that can lead to the irreversible impairment of DNA through methylation. TMZ monotherapy was first reported as a successful treatment for PC in 2006, which was then recommended by the ESE guideline as a first-line chemotherapy for PCs after the failure of standard therapy in 2018 (5). The recommended protocol of ESE guideline was using TMZ monotherapy (150–200 mg/m^2^ daily in consecutively repeated cycles (treatment given for 5 days every 28 days) in the case of documented tumor growth and suggested using the Stupp protocol [that is, concomitant administration of TMZ 75 mg/m^2^ daily and radiotherapy, followed by TMZ alone 150–200 mg/m^2^ daily (treatment given for 5 days every 28 days)] in the case of rapid tumor growth in patients who did not previously receive maximal doses of radiotherapy (5, 157, 158). In case reports and cohort studies, TMZ was reported to reduce tumor volume and hormonal levels in 47% of PC patients and induce partial or complete response in 71 of 149 patients in aggregate with populations ranging from 3 to 43 patients (5, 43, 51, 55, 126, 139, 140, 159–167). Our research indicated that the 2-year survival rate of PCs in patients who received TMZ treatment was increased by a minor degree compared to that of patients who did not receive TMZ but without statistical significance. It is suggested that the identification of responder and non-responder should be evaluated after three cycles of TMZ therapy, and the duration of continuing treatment for PC patients responding to first-line TMZ should be at least 6 months in total. MGMT methylation and DNA mismatch repair (MMR) proteins are known predictors of response to TMZ; therefore, it is strongly recommended that the precise selection of patients to receive TMZ treatment should be accomplished via the evaluation of MGMT (5, 131, 167–169). TMZ is often combined with radiotherapy, as it is a known radiosensitizer, and an ESE survey demonstrated that PC patients treated with concomitant chemoradiotherapy had a better tumor response (132). TMZ combined with radiotherapy may be a promising treatment for PC, as TMZ combined with whole-brain and spinal cord radiotherapy could induce the shrinkage of metastatic lesions. A new study indicated that TMZ was an effective medical treatment of PC, but was sometimes followed by tumor progression, and the co-administration with radiotherapy following the Stupp protocol may increase the progression-free survival rate (170). In addition to the above-mentioned treatments, other potential treatments include targeted therapies such as tyrosine kinase inhibitors against EGFR/HER2 such as lapatinib (171), the anti-VEGF antibody such as bevacizumab as a rescue treatment in combination with TMZ (5), the immune-checkpoint inhibitors (ICIs) such as inhibition of programmed death 1 (PD-1) and/or cytotoxic T-lymphocyte associated antigen 4 (CTLA-4) (172). The pharmacological mechanism of ICIs is based on the fact that PCs contain tumor­infiltrating lymphocytes and express PDL1, which is a potential predictor of response, as well as on emerging preclinical data on the efficacy of ICIs in murine models of PCs (158). Up to now, eight patients with PC were reported to be treated with ICIs, partial radiological response was observed in five patients (112, 113, 173), stable condition was observed in two patients (113, 174), and progressive condition was observed in one patient (113, 173), indicating that ICIs may be a new and promising treatment of PCs.

The mortality rate of reported PC patients was approximately 66% in 1 year and 80% in 8 years (142). Our research based on the reviewed literature demonstrated that 63 patients (49.2%) died as reported, 42 patients (32.8%) were alive at the time of publication, and 23 patients (18.0%) were ambiguous in their survival status. Among the patients who died, 37 patients died within 1 year of diagnosis, and 18 patients died within 4 years of diagnosis. The average survival time since the diagnosis of PC was 10.5 months, ranging from 6 months to 18 years. Though most PC patients died within 1 year after the metastasis was found, it was rare that some patients still showed survival greater than 5 years (35, 45, 48, 62). Due to the lack of large-scale clinical studies, there is still no universally recognized uniform standard for the mortality of PC patients.

## Conclusions

We described a 48-year-old male patient who was diagnosed with an atypical pituitary tumor initially and then was diagnosed with PC eventually after spinal cord metastasis was found, and the treatment course was illustrated as well. Furthermore, we summarized all the published case reports until now and provided a comprehensive review of the diagnosis, treatment, prediction, and clinical outcome of PC. We found that most PCs had ACTH/PRL-secreting tumors, Ki-67 ≥ 10%, and P53 positive, which may have the potential to predict the transformation from PAs to PCs; surgery excision combined with TMZ and radiotherapy is helpful to prolong the survival of PC patients. These results may enrich the knowledge about PCs and improve the clinical outcomes of this rare disease.

## Data Availability

The raw data supporting the conclusions of this article will be made available by the authors, without undue reservation.
